# Emerging Roles of the Gut Microbiome in Musculoskeletal Injury and Repair

**DOI:** 10.3390/microorganisms13092193

**Published:** 2025-09-19

**Authors:** Joseph L. Roberts, Connor C. Park

**Affiliations:** 1 College of Health Solutions, Arizona State University, Phoenix, AZ 85281, USA; 2 Biodesign Institute Center for Health Through Microbiomes, Arizona State University, Tempe, AZ 85281, USA; 3 Phoenix VA Health Care System, Phoenix, AZ 85004, USA; 4 Health Science Campus, Creighton University School of Medicine, Phoenix, AZ 85012, USA; connorpark@creighton.edu

**Keywords:** orthopedics, microbiome, fracture, trauma, osteoarthritis, probiotics

## Abstract

Over the past decade, significant attention has been directed toward understanding the role of the gut microbiome in health and disease. The gut microbiota, comprising a complex and diverse community of microorganisms, has been linked to numerous conditions, including metabolic disorders, gastrointestinal diseases, and inflammatory or autoimmune conditions. Recently, a growing body of evidence has revealed a compelling relationship between gut microbiota composition and musculoskeletal injury recovery, highlighting its potential as a novel therapeutic target. Musculoskeletal injuries, including fractures, post-traumatic osteoarthritis, and tendon or ligament injuries, commonly lead to changes in the community structure of the gut microbiota, intestinal permeability, and systemic inflammation, processes known to negatively influence tissue repair. Preclinical studies demonstrate that microbiota-targeted interventions, such as probiotics, prebiotics, and fecal microbiota transplantation, effectively restore gut barrier integrity, modulate inflammation, and normalize gut-derived metabolite profiles. Despite these promising findings, critical gaps remain in translating these effects into clinical practice, particularly regarding the mechanisms linking specific microbiota changes to improved musculoskeletal healing outcomes. Future research incorporating rigorous clinical trials, multi-omics analyses, and advanced predictive tools, including artificial intelligence and microbiome-informed digital twins, is urgently needed to fully harness the therapeutic potential of microbiome-based interventions in musculoskeletal injury recovery. This narrative review provides insights into our evolving understanding of the relationship between the gut microbiota and musculoskeletal injury and explores the potential of gut microbiota-targeted therapies for improved healing outcomes.

## 1. Introduction

The gut microbiome is the richest and most diverse microbial ecosystem in the human body, consisting of bacteria, viruses, fungi, and protozoa, with bacteria being the most abundant and extensively studied group. While defining what constitutes a healthy or optimal microbiome has proven to be a difficult endeavor, it is now generally well accepted that imbalances in the composition or metabolic functions of these microbial communities (i.e., dysbiosis) are associated with a wide array of diseases, including metabolic disease, gastrointestinal disorders, inflammatory conditions, and autoimmune diseases [1]. These associations have fueled widespread scientific interest in the microbiome across disciplines, contributing to an accumulating body of evidence over the past two decades that highlights the critical role of the gut microbiota in regulating nearly every aspect of human physiology and health [1].

More recently, the interplay between the gut microbiome and musculoskeletal conditions has received considerable attention. Musculoskeletal diseases and injuries are among the most common health conditions, affecting nearly half of adults aged 18 and older and more than 75% of those aged 65 and above [2,3]. Additionally, approximately 8% of emergency room visits in the US and Canada are attributed to musculoskeletal complaints [4,5], and orthopedic injuries are estimated to account for nearly 30% of emergency room and urgent care visits [6]. Given this substantial clinical and societal burden, along with emerging data connecting gut microbiota to musculoskeletal outcomes [7], the gut microbiome continues to be the subject of intense investigation as a new modifiable therapeutic target for musculoskeletal conditions.

While several prior reviews have explored the relationship between the gut microbiota and musculoskeletal conditions, most have focused on chronic diseases such as osteoporosis or general bone metabolism [8,9,10,11]. In contrast, few reviews have specifically addressed the effects of musculoskeletal injuries, such as fracture, joint injury, and tendon or ligament injuries, on the gut microbiota or examined the therapeutic potential of microbiota-targeted interventions in these contexts [7]. Emerging evidence also suggests that the gut microbiota may have differing effects depending on the type of musculoskeletal condition. In some cases, such as tendon injuries, the gut microbiota appear to promote tissue repair, whereas in others, such as post-traumatic osteoarthritis, they may contribute to disease progression. Recognizing this dual potential is critical for understanding when and how microbiota-targeted therapies may be beneficial. Therefore, the objective of this narrative review is to synthesize current evidence on the bidirectional relationship between the gut microbiota and musculoskeletal injury response, evaluate emerging microbiota-targeted therapies, and highlight key knowledge gaps and translational opportunities to guide future research and clinical application.

## 2. Brief Overview of the Gut Microbiome

Recognition of the gut microbiome as an essential contributor to human health dates back to ancient medical practices. Historical records indicate that traditional Chinese and Greek physicians employed fecal transplants as a treatment for gastrointestinal disorders, reflecting early recognition of the gut microbiota’s importance [12]. The modern concept of the microbiome was formally introduced by Joshua Lederberg in the early 21st century, defining it as the collective community of microorganisms inhabiting the human body [13]. Since then, scientific exploration has progressed toward increasingly precise and systematic approaches, including the detailed classification of microbial species within the gastrointestinal microbiome. Significant advancements have been achieved through large-scale initiatives, such as the National Institutes of Health’s Human Microbiome Project and the European Commission’s Metagenomics of the Human Intestinal Tract (MetaHIT) project [14]. Moreover, the advancement of sequencing and sampling technologies, particularly 16S ribosomal RNA sequencing, has revolutionized our understanding of gut bacteria by identifying previously unculturable and fastidious gut-resident microbial species [15]. In parallel, sophisticated experimental models, particularly germ-free animal models, have significantly enhanced our ability to study host–microbe interactions, providing deeper insights into the mechanisms linking microbiota to health and disease [16]. Fecal microbiota transplantation (FMT) studies, in which microbiota from diseased donors are transferred into healthy recipients, have also provided compelling evidence that the gut microbiota can exert causal effects on host physiology, rather than merely reflecting downstream consequences of disease [17]. These studies highlight the bidirectional nature of the host–microbiota relationship and underscore the active role of the gut microbiome in shaping health outcomes that extend beyond the gastrointestinal system.

**Figure 1:**
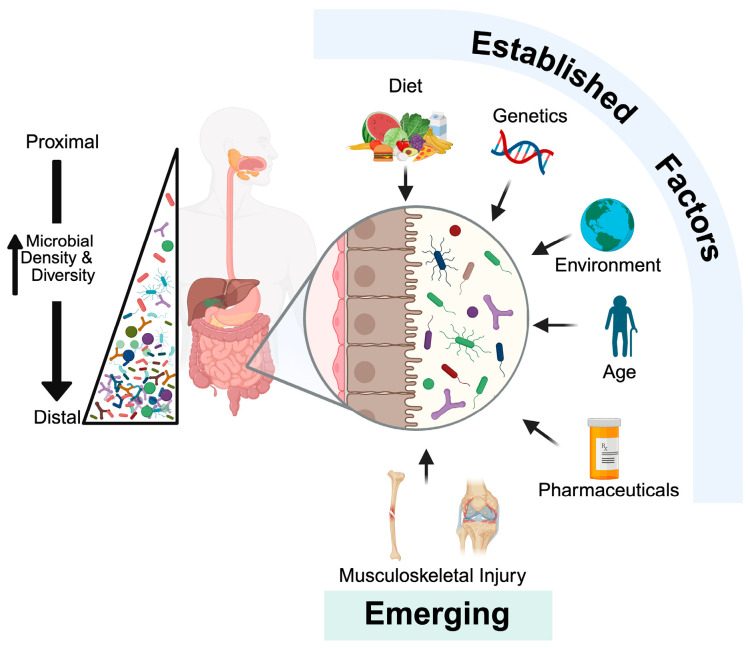
Microbiota composition is impacted by intrinsic and extrinsic factors. The gut microbiota increases in density and diversity from the mouth to the lower gastrointestinal tract. While several well-established factors are known to influence gut microbiota composition, trauma and musculoskeletal injuries are emerging as additional relevant modulators of microbial community structure. Created with BioRender.com.

Current estimates suggest that over 3000 unique bacterial species collectively inhabit the gastrointestinal system, which contains approximately 10^13^ microbial cells [18]. However, each individual likely harbors only a subset of these species, typically a few hundred at any given time [19]. The human gut microbiota is dominated by the phyla Firmicutes, Bacteroidetes, Actinobacteria, Verrucomicrobia, and Proteobacteria, with Firmicutes and Bacteroidetes accounting for approximately 90% of the microbial population [20]. While an adult’s gut microbiome remains relatively stable over time, its composition and diversity are highly plastic, continuously being shaped by a range of intrinsic and extrinsic factors (Figure 1) [21]. Importantly, this stability tends to gradually decline with advanced age, where increased inter-individual variability and altered phylum-level distributions are commonly observed [22]. Diet plays a predominant role in shaping microbial composition, while additional influences, such as host genetics, environmental exposures, pharmaceutical interventions, and sanitation practices, also contribute to microbiome development and stability [23,24]. Recent research has revealed the impact of trauma [25], as well as that of musculoskeletal injury, on gut microbiota composition, further elucidating the complex interplay between host physiology and gut microbiome dynamics [25,26,27]. These emerging relationships point to a potential role of the microbiota as a contributor to both systemic and local consequences of musculoskeletal injuries, which we discuss in detail below.

## 3. Impact of Musculoskeletal Injuries on Intestinal Function

Traumatic injuries significantly impact gastrointestinal physiology, often compromising the integrity of the gut barrier and leading to increased intestinal permeability, a condition more commonly referred to as “leaky gut.” This trauma-induced increase in intestinal permeability drives a systemic inflammatory response, facilitated partially by the paracellular transport of inflammatory bacterial proteins and products across the epithelial barrier [28,29,30]. A key mechanism underlying this inflammatory response involves the activation of toll-like receptor 4 (TLR4) signaling, the primary receptor for Gram-negative bacteria-derived lipopolysaccharides (LPS/endotoxins), and other pathogen-associated molecular patterns (PAMPs), which is essential for systemic inflammation following traumatic bone injury [30,31].

Initial evidence linking musculoskeletal injury to altered gut function dates back to pioneering preclinical studies in the 1990s. Using a murine model, these studies demonstrated that simple bone fractures increased intestinal permeability as early as 24 h after femur fracture [32] and increased T-cell populations within small intestinal Peyer’s patches [33]. Building on this work, our research, along with other studies, further demonstrated that femur fracture induces a lasting leaky gut phenotype that persisted up to seven days post-fracture, with gut permeability returning to pre-fracture levels by day 10 [27,34,35]. Further analysis revealed that several intestinal tight junction-associated genes, including *Cldn3*, *Cldn4*, *Cldn15*, *Olcn*, and *Tjp1*, as well as *Muc2*, were upregulated 10 days post-fracture within the small intestine [34]. This gene expression pattern corresponded to the restoration of gut barrier function and likely reflects an adaptive response by the intestine to repair the fracture-induced disruption of the barrier. Similar disruptions to the gut barrier and increased gut leakiness have been observed in joint injury models [36], indicating that musculoskeletal trauma negatively impacts intestinal function.

More recently, the gut microbiota has been recognized as a critical mediator of the intestinal response to musculoskeletal injury. Building on earlier findings of changes in T-cell populations [33], simple bone fractures increased expression of pro-inflammatory genes (*Il17a*, *Tnf*, *Il1b*, *Il6*) and the abundance of Th17 cells within intestinal Peyer’s patches in mice [35]. These Th17 cells were found to migrate from the intestine to the fracture callus, where they play an essential role in promoting fracture healing [35]. Importantly, these effects were dependent on the presence of Segmented Filamentous Bacteria (SFB) and an intact microbiota (i.e., unaltered, non-antibiotic-treated controls), as mice lacking SFB or with microbiota depleted by a broad-spectrum antibiotic cocktail did not exhibit the same intestinal inflammatory and T-cell responses [35]. This underscores the essential role of gut microbiota in regulating the intestinal and systemic responses to musculoskeletal injury.

## 4. Traumatic Musculoskeletal Injuries and Gut Microbiota Composition

A literature search was performed in PubMed and Google Scholar using combinations of keywords, including “gut microbiota,” “fracture,” “post-traumatic osteoarthritis,” “tendon,” “ligament,” and “musculoskeletal injury.” Both preclinical and clinical studies published in English were reviewed, with emphasis on those investigating microbiome composition and healing outcomes after musculoskeletal trauma. The sections below summarize recent evidence linking the gut microbiome to fracture healing, PTOA, and tendon/ligament injury.

### 4.1. Bone Fracture

Bone fractures represent common traumatic injuries, affecting approximately 179 million individuals worldwide in 2019 [37]. The systemic effects of fractures extend beyond the immediate site of injury, influencing physiological processes in various distal tissues, including significant alterations in gut microbiota composition. Our initial studies investigated fracture-induced microbiome shifts using 16S rRNA sequencing of fecal samples collected from young (12-week-old) C57BL/6J male mice before unilateral femoral fracture and again at 10 and 18 days post-injury [34]. Although alpha diversity remained consistent across all time points, distinct shifts in bacterial community composition were observed, characterized by a significant reduction in the relative abundance of bacteria belonging to the class Bacilli compared to baseline [34]. In a subsequent study conducted with aged (18-month-old) C57BL/6JN female mice, we again observed significant longitudinal changes in fecal microbiota composition following femoral fracture using metagenomic sequencing of fecal samples [27]. Surprisingly, significant alterations at the genus level emerged at three days post-injury, indicating that the gut microbiota rapidly responds to skeletal trauma. Furthermore, aged mice exhibited significant shifts in community structure at day 14 post-fracture, marked by increased alpha diversity [27].

To explore potential age-related differences further, we assessed gut microbiota responses to femoral fracture in young (3-month-old) and middle-aged (15-month-old) C57BL/6J female mice [26]. Consistent with previous literature [38,39,40,41,42,43], baseline gut microbiota compositions differed significantly between age groups. At day 3 post-fracture, both young and middle-aged mice displayed a significant shift in the composition of the gut microbiota, with the middle-aged mice showing a marked increase in Enterobacteriaceae, a family containing several pathogenic species. Moreover, the middle-aged mice experienced another distinct shift in microbiota structure at day 14 post-fracture, a pattern not observed in younger mice, potentially indicating age-related differences in microbiota resilience [26]. At the genus level, young mice showed an increased relative abundance of health-promoting *Bifidobacterium* at days 10 and 14 post-fracture, along with a sustained decrease in *Lactobacillus* at days 10, 14, and 18. In contrast, these specific changes were not evident in middle-aged mice. Instead, middle-aged mice exhibited an increase in *Muribaculum* and a decrease in *Ligilactobacillus* at day 3 post-fracture. Collectively, these observations indicate that bone fractures rapidly induce a shift in microbiota community structure, highlighting critical age-dependent differences in gut microbial responses to trauma and underscoring the complexity of host–microbiome interactions following bone injury. However, the underlying reasons for this injury-associated shift in the gut microbiota remain unclear, and it is unknown whether this shift reflects a form of dysbiosis or a normal physiological response to trauma. The functional significance of these changes also warrants further investigation. One potential contributing factor is the significant reduction in food intake commonly observed in the immediate post-injury period. In our studies, mice exhibited an approximately 40–50% decrease in food consumption within the first 48 h post-fracture (unpublished data), which may partly explain the observed changes in microbiota composition. Other outstanding questions include determining whether specific microbial taxa or individual enterotypes directly influence fracture healing outcomes, and if microbiota-driven inflammatory or immunological pathways mediate bone repair processes. Clarifying these questions will provide critical insights into host–microbiome interactions, potentially identifying new therapeutic targets to accelerate fracture repair.

### 4.2. Post-Traumatic Osteoarthritis

Osteoarthritis (OA) is a degenerative joint disease characterized by the breakdown of articular cartilage, synovial inflammation, thickening of subchondral bone, formation of osteophytes, and hypertrophy of the joint capsule. Collectively, these changes contribute to symptomatic pain and functional impairment [44]. OA is a prevalent condition, affecting approximately 7% of the global population, and remains the leading cause of disability among older adults [45,46]. Although often considered a natural consequence of aging, symptomatic OA is exacerbated by various comorbidities, including obesity, diabetes, and chronic pro-inflammatory conditions [47]. Post-traumatic osteoarthritis (PTOA) is a distinct subset of OA that accounts for approximately 12% of all OA and arises independently of age, developing after traumatic injury to the joint [47,48]. Such injuries frequently result from recreational activities involving acute mechanical loading events that damage the soft tissues of the knee joint [48]. Due to the complex, multifactorial nature of both OA and PTOA, studying their origins and progression in human subjects presents significant challenges. Translational animal models serve as essential tools, providing critical insights into the underlying pathophysiological processes and enabling detailed exploration of disease initiation, progression, and potential therapeutic targets [49]. Most preclinical OA studies utilize PTOA models of knee joint OA, which are either invasively or non-invasively initiated. Common invasive approaches include anterior cruciate ligament (ACL) transection (ACLT), destabilization of the medial meniscus (DMM), and meniscal/ligamentous injury (MLI). Non-invasive approaches include tibial compression overload to rupture the ACL and targeted joint impact [49].

A recent conference abstract reported changes in the gut microbiota community structure of conventionally raised mice following DMM surgery. Compared to uninjured controls, animals that developed PTOA exhibited only modest microbial shifts in cecal samples via metagenomic sequencing [50]. Specifically, PTOA mice exhibited an increased abundance of bacterial genera such as *Lachnospiraceae* and *Anaeroplasma*, alongside a reduction in *Bifidobacterium pseudolongum*. In contrast, a separate study using 16S rRNA gene sequencing identified more pronounced shifts in gut microbiota composition eight weeks after DMM [51]. Compared to sham-operated controls, mice with PTOA demonstrated a significant increase in the relative abundance of the phyla Firmicutes and Actinobacteria and a significant reduction in the relative abundance of Bacteroidota, Verrucomicrobiota, Desulobacterota, Proteobacteria, and Cyanobacteria [51].

Using murine models, several studies have identified a clear link between gut microbiota and PTOA pathogenesis [52,53,54,55,56]. Initial studies demonstrated that germ-free C57BL/6J mice subjected to DMM surgery exhibited significantly less cartilage damage, proteoglycan loss, and osteophyte formation compared to specific pathogen-free controls, highlighting the critical role of microbiota in PTOA development [53,54]. A subsequent study employing a non-invasive ACL rupture model further supported these findings, revealing that germ-free mice experienced reduced severity of PTOA-related bone loss compared to pathogen-colonized controls [55]. Using the same ACL rupture model, depleting the microbiome of mice using an antibiotic cocktail (ampicillin and neomycin) prior to joint injury significantly slowed cartilage degradation and decreased joint inflammation [52]. These findings support the hypothesis that gut microbiota are essential contributors to PTOA progression. These findings also support the “two-hit” hypothesis, in which systemic inflammation triggered by gut microbiota-derived PAMPs or other inflammatory conditions constitutes the first hit, while joint injury represents the second. Together, these insults synergistically amplify inflammatory responses to promote the development of PTOA [53]. An elegant study sought to test this hypothesis by transplanting fecal microbiota from human donors categorized into three groups: (1) donors without OA, (2) donors with symptomatic knee OA (Kellgren/Lawrence grade III or IV), and (3) donors with symptomatic knee OA combined with metabolic syndrome [56]. Germ-free C57BL/6J mice receiving these transplants were subsequently subjected to MLI surgery to model joint trauma [56]. Consistent with earlier findings, germ-free mice demonstrated reduced OA severity compared to specific pathogen-free controls [56]. Mice receiving FMT from donors with metabolic syndrome combined with symptomatic knee OA exhibited significantly aggravated PTOA severity [56]. Further microbial analysis identified positive correlations between higher OARSI histological scores (indicating worse OA) and increased abundance of the bacterial genera *Candidatus_Stoquefichus*, *Faecalibacterium*, *Tyzzerlla_4*, and *Fusobacterium* [56]. Conversely, a negative correlation was observed with beneficial genera, including *Erysipelatoclostridium*, *Eubacterium*, *Marvinbryantia*, *Bacteroides*, and *Ruminococcaceae_UCG-013* [56].

Additionally, sex-specific differences have been identified, with microbiota composition influencing the susceptibility and severity of PTOA differently in male and female mice [57]. Male mice receiving female microbiota had improved OA outcomes, while female mice receiving male microbiota developed more severe PTOA after DMM. 16S rRNA sequencing revealed that certain taxa, including *Lactobacillus*, *Adlercreutzia*, *rc4_4*, and *Sutterella*, were negatively associated with OA severity, whereas members of the *Clostridiales* order were positively correlated [57]. Collectively, these studies indicate that gut microbiota can exacerbate joint trauma, thereby significantly influencing PTOA pathogenesis.

### 4.3. Tendon and Ligament Injury

Injury to tendons and ligaments are estimated to account for as high as 50% of all musculoskeletal injuries annually [58]. These injuries also have a significant negative impact on patients’ quality of life. Although most injuries to tendon and ligaments heal without surgical intervention, their inherently poor blood supply leads to a prolonged healing process. Healing often results in the formation of inferior scar tissue, which may require years to remodel into more functional tissue. Consequently, many severe cases necessitate grafting or surgical interventions [59]. The acute inflammatory response following injury initiates callus formation in tendons and ligaments, a process heavily dependent on a well-coordinated immune response [60]. However, the remodeling capacity of these soft tissues is markedly less efficient compared to bone following fracture [61]. Given the substantial preclinical evidence demonstrating the critical role of the gut microbiome in bone remodeling, recent studies have begun exploring how gut microbiota may similarly influence tendon and ligament healing.

Although research in this area remains limited, emerging evidence indicates that the composition of the gut microbiome plays a critical role in regulating ligament and tendon repair following traumatic injury. Platelet-rich plasma (PRP) injections have been considered a potential therapy for improving the healing of these structures, yet their success in clinical settings has been variable [62]. Dietrich et al. proposed that this inconsistency could be attributed to differences in the gut microbiome and its influence on immune-mediated healing responses [63]. Their work revealed that specific pathogen-free rats exhibited no improvement in Achilles tendon mechanical properties with PRP treatment, whereas rats colonized with the pathogenic bacterium *Staphylococcus aureus* showed enhanced tendon healing, characterized by increased CD8+ T-cell levels [63]. Similarly, the effectiveness of dexamethasone, an anti-inflammatory steroid, was found to vary depending on the microbiome status [64]. More recently, Dietrich-Zagonel et al. demonstrated that the gut microbiome also influences tendon healing responses to mechanical loading after Achilles tendon transection in rats [60]. These findings provide important insights into potential gut microbiome–tendon interactions, highlighting the necessity for further studies to clarify the precise role of microbiota in tendon and ligament repair following traumatic injuries.

## 5. Targeting the Gut Microbiota After Musculoskeletal Injury

The gut microbiota has emerged as a promising therapeutic target for various acute and chronic diseases due to its inherent accessibility and responsiveness to targeted interventions. Microbiota-targeted therapeutic strategies are highly accessible and simple in nature and typically involve consumption of probiotics, prebiotics, or synbiotics (combined prebiotic and probiotic formulations), as well as FMT, under medical supervision. Each approach aims to modulate the gut microbiome composition and functionality, thereby potentially enhancing healing, reducing inflammation, and promoting overall musculoskeletal recovery. In this section, we provide a detailed overview of current research on gut microbiota-targeted therapies specifically applied in the context of musculoskeletal injury, evaluating their efficacy, underlying mechanisms, and future therapeutic potential. A literature search was performed in PubMed and Google Scholar using combinations of keywords, including “probiotics,” “prebiotics,” “fecal microbiota transplant,” “gut microbiota,” “fracture,” “post-traumatic osteoarthritis,” “tendon,” “ligament,” and “musculoskeletal injury.” Both preclinical and clinical studies published in English were screened to identify evidence on how these interventions influence recovery after musculoskeletal injury. We summarize key findings in Table 1, which presents a non-exhaustive selection of studies relevant to gut microbiota-targeted interventions in musculoskeletal injury, identified based on their relevance to the focus of this narrative review.

### 5.1. Probiotics

Probiotics are live microorganisms which, when administered in adequate amounts, provide a health benefit to the host [65]. Probiotics are frequently consumed through food products such as yogurt or as dietary supplements in non-food forms. According to data from the National Health and Nutrition Examination Survey (NHANES), approximately 5% of the U.S. population regularly consumes probiotic dietary supplements, highlighting their growing popularity [66]. Most probiotic species belong to genera such as *Bifidobacterium*, *Saccharomyces*, *Streptococcus*, *Enterococcus*, *Escherichia*, *Bacillus*, and the family *Lactobacillaceae*. These health-promoting bacteria interact with host physiology through various mechanisms, including competitive exclusion of pathogenic bacteria, enhancement of the intestinal mucosal barrier integrity, modulation of immune responses, and the production of beneficial bioactive metabolites such as short-chain fatty acids [67]. Importantly, while probiotics universally offer health benefits, their mechanisms of action and efficacy can vary considerably, often being species- or even strain-specific [68]. Initially developed to address gastrointestinal disorders, probiotics are now increasingly explored for their therapeutic potential in a range of acute and chronic conditions, including musculoskeletal injuries.

The return to daily activities is a fundamental measure of recovery following an acute musculoskeletal injury. The limited clinical studies focusing on probiotics and musculoskeletal injury recovery have focused predominately on symptomatic pain and functionality assessments following fracture. One of the most extensively studied probiotic strains is *Lactobacillus casei* Shirota, recently renamed *Lacticaseibacillus paracasei* strain Shirota, which is commonly consumed within the probiotic drink Yakult. Clinical studies investigating this strain in patients with acute fractures have reported a faster self-reported return to functional status, significant improvements in range of motion, and, notably, confirmed its safety even at higher dosages [69,70]. Although these studies did not directly measure fracture healing, the observed improvements in functionality suggest a potential benefit of probiotic consumption in accelerating fracture recovery.

Recent preclinical investigations are beginning to clarify how specific probiotic strains can modulate fracture repair [27,34,71,72]. In our initial work, *Bifidobacterium adolescentis* given for two weeks before femoral fracture accelerated cartilage-to-bone transition within the callus in male C57BL/6J mice [34]. *B. adolescentis* supplementation also decreased systemic cytokines levels (IL-16 and IL-6), suppressed pro-inflammatory cytokine gene expression in bone marrow and colon, and preserved gut barrier integrity by upregulating tight junction genes, thereby decreasing intestinal permeability and serum endotoxin levels [34]. Liu and colleagues extended these findings by administering *Akkermansia muciniphila*, *Lactobacillus gasseri*, or PBS vehicle control to female C57BL/6 mice after femur fracture. Both strains accelerated callus maturation, improved mechanical strength, and attenuated gut permeability and systemic inflammation [72]. Benefits of *Bifidobacterium* supplementation were also observed in aged animals. Daily *Bifidobacterium longum* given to 18-month-old female C57BL/6 mice accelerated bony callus formation, enhanced biomechanical properties, dampened systemic inflammation, and maintained intestinal epithelial tight junction integrity [27]. Importantly, a recent study asked whether oral probiotic treatment initiated before or after fracture is needed for optimal healing benefits [71]. To test this, young male C57BL/6 male mice were given the proprietary VSL #3 probiotic blend consisting of eight probiotic strains (*Lactobacillus acidophilus*, *Lactobacillus plantarum*, *Lactobacillus casei*, *Lactobacillus delbrueckii* subspecies *bulgaricus*, *Bifidobacterium breve*, *Bifidobacterium longum*, and *Bifidobacterium infantis* and *Streptococcus salivarius* subspecies *thermophilus*) for 5 weeks prior to fracture or for 4 weeks after fracture [71]. Pre- and post-fracture supplementation with the probiotic blend led to significant improvements in the biomechanical properties of the healed femur; however, only the mice that received the probiotic prior to fracture displayed changes in callus inflammatory gene expression and improvements in callus bone microarchitecture [71]. Current preclinical evidence shows that both single-strain probiotics and multi-strain blends can enhance secondary fracture healing when initiated before or after bone fracture. Moreover, a common mechanism emerges in that probiotics preserve intestinal barrier integrity after fracture and dampen the accompanying systemic inflammatory response. These findings position probiotics as an adjunct therapy for improving fracture repair and underscore the need for additional rigorous clinical trials to validate their benefits in patients recovering from fractures.

Probiotics also hold promise in protecting joint integrity by mitigating synovial inflammation [73]. Specific species supplementation, such as *Saccharomyces boulardii* and *Lacticaseibacillus paracasei* Shirota, result in significant improvements in pain scores and decreased C-reactive protein in humans with knee OA [74,75]. Complementary preclinical studies further support the protective effects of probiotics on joint structure after injury. Several probiotic species, including *Lactobacillus acidophilus*, *Clostridium butyricum*, and *Streptococcus thermophilus*, have shown chondroprotective effects in mouse and rat models of PTOA, evidenced by reduced cartilage degeneration, improved OARSI scores, and enhanced weight-bearing capacity [76,77,78,79,80]. Mechanistically, these beneficial effects appear to arise primarily from attenuation of systemic and local (joint and synovial) inflammation, highlighting the promise of probiotics as adjunctive therapies to mitigate PTOA.

### 5.2. Prebiotics

Prebiotics are functional, plant-derived food components that promote host health by supporting the growth of beneficial gut microbes. Prebiotics are typically consumed as low molecular weight carbohydrates, including oligosaccharides and galactooligosaccharides. As a subset of dietary fiber, prebiotics fall within a nutrient category for which only 5% of Americans meet the recommended daily allowance (RDA) [81]. Dietary fiber is broadly categorized into two types: insoluble and soluble. Insoluble fiber primarily aids digestive health by increasing stool bulk and promoting regular bowel movements. In contrast, soluble or fermentable fiber serves as a substrate for gut microbiota, which metabolize these fibers into bioactive compounds such as short-chain fatty acids (SCFAs) that play key roles in maintaining gut and systemic health. While prebiotic fibers are found naturally in fruits and vegetables, food manufacturers are increasingly adding prebiotics (e.g., inulin) to foods during processing to increase the fiber content.

While extensive research has focused on unraveling the effects of prebiotics on bone homeostasis its impact on musculoskeletal injury remains largely unexplored [82]. This represents a critical gap in our understanding of how dietary interventions might modulate healing outcomes following musculoskeletal injury. Emerging evidence from clinical and preclinical studies suggests that prebiotics may confer functional benefits in the context of OA. A recent clinical study found that oligofructose-enriched inulin prebiotic supplements improved performance in the timed-up-and-go test, 40 m fast paced walk test, and hand grip strength test, as well as a trend towards improvement in knee pain in individuals with obesity and knee OA [83]. Preclinical findings further support the potential therapeutic role of prebiotics in joint injury. Diets enriched with oligofructose (10% *w*/*w* Orafti P95) attenuated cartilage degradation and slowed PTOA progression following DMM surgery in both lean and obese mice [36,84]. Similarly, high-fiber diets containing 20% plant polysaccharides protected against ACLT-induced PTOA in male rats [85]. Mechanistic studies suggest that prebiotic supplementation enhances gut barrier integrity by upregulating tight junction proteins, leading to reduced systemic LPS levels and inflammation [36]. Unbiased fecal metabolomic profiling further revealed that PTOA alters the abundance of over 200 fecal metabolites, while prebiotic treatment normalized levels of several metabolites associated with joint pathology to levels observed in uninjured controls [36]. Collectively, these findings underscore the potential of prebiotics to preserve joint integrity and modulate disease progression following injury. However, whether similar benefits extend to other forms of musculoskeletal trauma, such as fractures or tendon/ligament tissue injuries, remains an important and largely unexplored area of research.

### 5.3. Fecal Microbiota Transplants (FMT)

FMT involves transferring a complete microbial community from a healthy donor to a recipient with disease, and has shown remarkable success in treating *Clostridium difficile* infection and symptoms related to autism spectrum disorders [86,87]. Interest in FMT has expanded rapidly to other areas of medicine, including conditions associated with gut dysbiosis and its potential influence on musculoskeletal health. However, clinical implementation of FMT remains limited due to uncertainties regarding optimal dosing, standardized manufacturing, and appropriate storage practices [87]. The impact of FMT on musculoskeletal healing outcomes, particularly following acute injury, is not yet well understood. A recent study utilizing the DMM PTOA model demonstrated promising results, showing that FMT from MRL/MpJ “super healer” mice, which exhibit resistance to PTOA, into conventionally raised C57BL/6J mice significantly reduced osteoarthritis severity [54]. Specifically, recipient mice showed decreased OARSI scores, synovitis scores, and osteophyte formation, highlighting the potential of FMT to influence healing and disease outcomes following musculoskeletal injury [54]. Similar to probiotic therapy in the fracture literature, microbiota from the super healer mice decreased gut permeability and peripheral immune cell populations [54]. Overall, these early findings indicate that FMT may hold significant promise as a novel therapeutic approach to modulate gut microbiota and potentially improve healing outcomes following joint injury. However, additional studies are needed to explore its applicability to other musculoskeletal injuries.

**Table 1:** Representative studies on gut microbiota-targeted interventions after musculoskeletal injury. Up arrow means increased. Down arrow means decreased.

Injury Model	Subjects	Intervention	Key Results	Ref.
Fracture				
Closed unilateral femur fracture	C57BL/6J Male (10 weeks old)	*Bifidobacterium adolescentis* (ATCC 15703); 1 × 10^8^ CFU 5 days per week oral gavage two weeks prior to fracture until sacrifice	↑ Cartilaginous callus remodeling ↓ Gut permeability ↓ Inflammation	[34]
Open osteotomy femur fracture	C57BL/6 Female (8 weeks old)	*Akkermansia muciniphila* (ATCC BAA-835); 8 × 10^8^ CFU twice per week oral gavage after fracture	↑ Callus bone ↑ Biomechanical properties ↓ Gut permeability ↓ Inflammation	[72]
Open osteotomy femur fracture	C57BL/6 Female (8 weeks old)	*Lactobacillus gasseri* (ATCC 33323); 8 × 10^8^ CFU twice per week oral gavage after fracture	↑ Callus bone ↑ Biomechanical properties	[72]
Closed unilateral femur fracture	C57BL/6JN Female (18 months old)	*Bifidobacterium longum* (ATCC 15707); 1 × 10^8^–1 × 10^9^ CFU daily oral gavage two weeks prior to fracture until sacrifice	↑ Callus bone ↑ Biomechanical properties ↓ Gut permeability ↓ Inflammation	[27]
Open osteotomy Femur Fracture	C57BL/6 Male (11 weeks old)	VSL#3 probiotic blend; 1 × 10^9^ 5 days per week for 5 weeks prior to fracture or for 4 weeks after fracture	Pre-treatment: ↑ callus bone and mechanical properties. Post-treatment: ↑ callus bone and mechanical properties.	[71]
Post-traumatic Osteoarthritis (PTOA)				
DMM	C57BL/6J (19 weeks old)	Prebiotic-supplemented high-fat diet containing 10% *w*/*w* Beneo-Orafti Orafti P95 Oligofructose	↓ Cartilage damage	[84]
ACLT	Sprague-Dawley Rats	Diets containing 20% plant polysaccharides	↓ Cartilage damage ↓ Systemic inflammation ↓ Pain	[85]
DMM	C57BL/6 Male (9 weeks old)	Prebiotic-supplemented high-fat diet containing 10% *w*/*w* Beneo-Orafti Orafti P95 Oligofructose	↓ OARSI scores ↓ Osteophyte size ↓ Gut permeability	[36]
PMM	C57BL/6J Female (11 weeks old)	*Lactobacillus acidophilus* (ATCC 4356); 3 × 10^9^ CFU twice per week oral gavage after PMM	↓ OARSI scores ↓ Pain	[77]
ACLT	Sprague-Dawley Rats Male (8 weeks old)	*Lactobacillus plantarum* GKD7 (5 × 10^10^ CFU/kg bw) daily oral gavage after ACLT	↓ OARSI scores ↑ Weight bearing ↓ Joint inflammation	[79]
ACLT	Male Wistar Rats (8 weeks old)	*Streptococcus thermophilus* TCI633 (5 × 10^9^, 5 × 10^10^, or 5 × 10^11^ CFU/kg/day) daily oral gavage after ACLT	↓ OARSI scores ↓ Knee swelling ↓ Mechanical allodynia ↓ Cartilage apoptosis	[80]
ACLT	Sprague-Dawley Rats (8 weeks old)	*Clostridium butyricum* GKB7; daily oral gavage after ACLT	↓ OARSI scores ↑ Weight bearing ↓ Joint inflammation	[78]
DMM	C57BL/6 Male (8-weeks-old)	*Lacticaseibacillus paracasei* 8700:2 (DSM13434), *Lactiplantibacillus plantarum* HEAL9 (DSM15312), *Lactiplantibacillus plantarum* HEAL19 (DSM12313) in drinking water (10^8^ CFU/mL) after DMM	↓ OARSI score at medial femoral condyle ↑ Trabecular bone in femoral epiphysis	[88]
ACLT	C57BL mice (6–8 weeks-old)	*Streptococcus thermophilus* CICC 6222/ATCC 19258 or *Lactobacillus pentosus* CICC 24202 or combined; 2 × 10^10^ CFU/kg daily oral gavage starting two weeks prior to ACLT	↓ OARSI scores ↓ Joint inflammation	[76]

### 5.4. Central Mechanisms of Microbiota-Targeted Interventions

**Figure 2:**
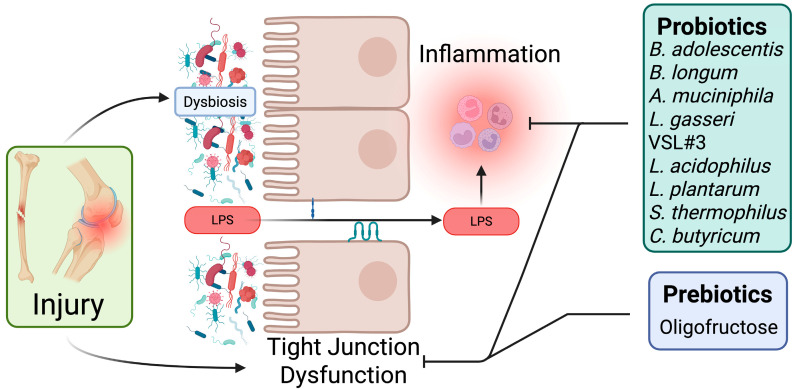
Shared mechanisms of action underlying microbiota-targeted interventions. Musculoskeletal injury alters gut microbiota composition and compromises intestinal barrier integrity, resulting in increased gut permeability and systemic inflammation. Preclinical studies have shown that probiotics and prebiotics can reduce intestinal permeability and inflammation following fracture and post-traumatic osteoarthritis. Created with BioRender.com.

Current evidence identifies several shared mechanisms underlying the therapeutic effects of microbiota-targeted interventions following musculoskeletal injury, irrespective of the specific injury type (i.e., fracture, joint, tendon) or therapeutic (i.e., probiotic, prebiotic) approach (Figure 2). A central mechanism common to probiotics, prebiotics, and other microbiota-targeted therapies is the restoration of gut barrier integrity. Musculoskeletal injuries induce increased intestinal permeability due to the disruption and dysregulation of epithelial tight junction proteins [34,36]. This compromised gut barrier permits systemic translocation of bacterial components, triggering heightened systemic inflammation that can impair tissue repair and recovery. Probiotic supplementation from multiple genera has demonstrated efficacy in restoring gut barrier function by upregulating the expression of tight junction proteins, thereby reducing gut permeability, systemic endotoxemia, and subsequent inflammation, ultimately improving musculoskeletal healing outcomes [27,34,72]. Similarly, prebiotic interventions enhance gut barrier integrity and decreased serum endotoxin levels after joint injury [36].

Modulation of inflammation is another mechanism shared across microbiota-targeted interventions. While acute inflammation is necessary for initiating tissue repair, sustained elevation of systemic inflammation following injury can negatively impact healing and joint integrity [89,90]. Microbiota-based therapeutic strategies consistently demonstrate anti-inflammatory properties, reducing both local and systemic inflammatory responses [27,34,36,71,72,76,77,79]. These anti-inflammatory effects are associated with improved fracture repair outcomes, and reduced cartilage degradation and synovitis in PTOA models. However, despite consistent findings regarding improvements in gut barrier function and systemic inflammation, the precise molecular mechanisms underpinning these benefits remain unclear. Outstanding questions include whether these anti-inflammatory effects primarily result from improvements in intestinal barrier integrity, direct modulation of gut-associated immune cell populations, or a combination of these and other yet-to-be-identified pathways. Further investigation into these mechanistic pathways is essential to fully understand and leverage microbiota-based therapeutic strategies.

One emerging mechanistic area is gut microbiota-derived metabolites, or postbiotics, which exert effects both locally within the gastrointestinal tract and systemically at sites of injury. The gut microbiota is a significant contributor to host metabolism by producing or modifying an estimated 10% of the metabolites in blood, as well as more than 50% of those in feces and urine [91,92]. Many of these metabolites exhibit potent immunomodulatory and anti-inflammatory properties that potentially support tissue regeneration [93]. Unbiased fecal metabolomics studies reveal significant alterations in gut-derived metabolites following PTOA [55], and interventions such as prebiotic supplementation have demonstrated the ability to restore metabolite profiles toward those observed in uninjured controls [36]. Experimental studies evaluating microbiota-derived metabolites in PTOA models have demonstrated that capsiate and γ-aminobutyric acid (GABA) exhibit remarkable chondroprotective effects [51,76]. While probiotic bacteria have inherent metabolic activity, they also interact with the indigenous microbiota to influence the collective metabolism of the microbiome [94]. In both fracture and PTOA models, probiotic supplementation consistently alters gut microbiota composition, likely shifting microbial metabolic outputs [27,34,77]. Further mechanistic studies are needed to clarify whether the therapeutic benefits arise from metabolites produced directly by the administered probiotic strain or from the metabolic activity of the restructured gut microbiota, as well as the roles of these metabolites in the context of musculoskeletal injuries. Moreover, clinical studies are urgently needed to determine whether the exciting benefits of microbiota-targeted therapies observed in preclinical models translate to humans. Ultimately, advancing our understanding of these shared microbiota-driven mechanisms holds substantial promise for developing targeted, personalized therapeutic strategies that improve clinical outcomes for patients after musculoskeletal injury.

## 6. Future Directions

### 6.1. Defining the Optimal Microbiota Composition for Musculoskeletal Repair

Many patient-specific factors influence musculoskeletal healing, including nutritional status [95], age [96], and comorbidities [97,98]. As reviewed above, there is now a wide body of literature indicating that a patient’s gut microbiota is also a key contributing factor to repair processes that should be considered. Currently, standard medical care does not consider the gut microbiota. However, there is currently no clearly defined microbiota composition or specific “healthy microbiome” profile identified for optimal musculoskeletal healing outcomes. Future research should prioritize identifying microbial signatures associated with enhanced healing, reduced systemic inflammation, and improved functional recovery. Additionally, it is important to determine if beneficial microbial profiles vary according to age, sex, or specific injury types and how these differences influence injury repair and disease progression. As discussed above, the literature on PTOA indicates that gut microbiota from male and female mice differently impact disease severity [57], but it remains unclear if similar sex-specific effects of the microbiota occur in other musculoskeletal injuries, such as fracture or tendon injury. Further, understanding the distinct microbiota compositions in aged populations and their potential influence on musculoskeletal healing outcomes warrants dedicated investigation, as they commonly experience fragility fractures. In addition to demographic factors, the impact of the gut microbiota appears to also vary depending on the type of musculoskeletal injury. For instance, in acute injury to tendon and bone, the microbiota may facilitate healing through modulation of inflammation and immune cell activity that is critical for initiating repair. In PTOA, which develops gradually following joint injury, persistent low-grade inflammation and systemic immune priming, although less pronounced than the acute response observed after fracture, may attenuate or even negate these beneficial effects. These condition-specific differences may be driven by variability in gut microbiota composition or the inflammatory response to the injury. Clarifying these relationships, including how age, sex, injury type, and immune context shape the role of the microbiota, will support the development of personalized therapeutic strategies that target gut microbes to optimize recovery following musculoskeletal injuries.

### 6.2. Artificial Intelligence and Predictive Modeling of Healing Outcomes Using Microbiome Data

The rapid advancements in artificial intelligence (AI), particularly machine learning (ML), present a promising frontier for microbiome research by offering powerful tools to understand and predict musculoskeletal healing outcomes based on gut microbiota composition [99,100]. AI-driven analysis of complex microbiome datasets can uncover previously unrecognized connections between gut microbial communities and musculoskeletal health, facilitating the discovery of novel biomarkers predictive of healing trajectories. Preclinical studies have demonstrated correlations between specific microbial taxa or microbiota profiles and differential healing outcomes, exemplified by varied fracture healing responses in mice containing SFB [35] or tendon healing influenced by *Staphylococcus aureus* [63,64]. Leveraging ML algorithms that effectively integrate multi-omics data could enable personalized therapeutic strategies tailored precisely to an individual’s unique microbiome composition, potentially shortening recovery periods and enhancing overall quality of life. One particularly promising direction is the creation of personalized “digital twin” virtual models that integrate a patient’s microbiome and clinical data to simulate and predict how specific therapies might influence healing outcomes [101]. Such digital twins would allow clinicians to assess the efficacy of microbiome-based interventions such as probiotics or prebiotics virtually, optimizing treatment strategies before implementation [102]. This personalized approach is essential, as an individual’s indigenous microbiota can influence colonization resistance to probiotics [103] and modulate physiological responses to prebiotic fibers [104]. However, successful clinical translation of these technologies requires rigorous validation through prospective studies to assess sensitivity, specificity, and practical utility of microbiome-derived biomarkers and predictive models in musculoskeletal healing. Future research should prioritize identifying and validating microbial signatures associated with optimal or impaired healing, enabling early and targeted microbiome-based interventions guided by non-invasive fecal sampling. The continued evolution and integration of AI methodologies and digital twin platforms into microbiome and musculoskeletal sciences could soon make personalized, microbiome-informed interventions a routine component of patient care.

## 7. Conclusions

Interest in the gut microbiota has grown substantially among scientists, clinicians, and the general public, driven by accumulating body of evidence linking the gut microbiota to nearly every aspect of human health. Recent studies increasingly underscore the compelling and complex relationship between musculoskeletal injury and gut microbiota composition, identifying the microbiome as a promising therapeutic target. Mechanistically, the gut microbiota can significantly influence systemic inflammation, tissue integrity, and healing responses after a musculoskeletal injury, supporting the rationale for microbiota-targeted therapeutic interventions. While the relationship between gut microbiota and bone health in clinical populations has received more attention than its role in musculoskeletal injury, both areas remain underexplored, and our understanding of how the microbiota influences healing processes in humans is still limited. Current preclinical studies strongly support a microbiome–musculoskeletal injury connection, laying the essential groundwork for translating findings into well-designed clinical studies. To fully leverage the therapeutic potential of microbiome-based strategies, future research must address remaining knowledge gaps, particularly regarding the causal relationships and specific microbial mechanisms involved in healing processes. Rigorous clinical trials and integrative multi-omics studies, combined with advancements in artificial intelligence and machine learning, are now needed to design, validate, and personalize microbiome-based therapies for musculoskeletal repair. Successfully harnessing the gut microbiome, therefore, holds great promise for transforming orthopedic practice and substantially improving long-term quality of life for patients.

## Data Availability

No new data were created or analyzed in this study. Data sharing is not applicable to this article.

## Abbreviations

The following abbreviations are used in this manuscript:

ACLT	Anterior cruciate ligament transection
AI	Artificial intelligence
DMM	Destabilization of the medial meniscus
FMT	Fecal microbiota transplant
LPS	Lipopolysaccharide
ML	Machine Learning
MLI	Meniscal/ligamentous injury
OA	Osteoarthritis
OARSI	Osteoarthritis Research Society International
PAMP	Pathogen-associated molecular patterns
PMM	Partial Meniscectomy Model
PRP	Platelet-Rich Plasma
PTOA	Post-traumatic osteoarthritis
SFB	Segmented Filamentous Bacteria
TLR4	Toll-like receptor 4
